# Integrated Physical Therapy in a Unique Case of Holstein-Lewis Fracture With Radial Palsy: A Case Report

**DOI:** 10.7759/cureus.57117

**Published:** 2024-03-28

**Authors:** Aditi Akhuj, Pratik Phansopkar

**Affiliations:** 1 Musculoskeletal Physiotherapy, Ravi Nair Physiotherapy College, Datta Meghe Institute of Higher Education & Research, Wardha, IND

**Keywords:** distal humerus fracture, case report, physiotherapy, robotic gloves, radial nerve palsy, holstein-lewis fracture

## Abstract

The term "Holstein-Lewis fracture" describes a spiral fracture that occurs in the shaft of the humerus at its distal third, which has been linked to radial nerve palsy in adults, and operative treatment is the preferred method of treating the trapped nerve at the fracture site. This paper describes a clinical case involving a 20-year-old male patient demonstrating a humeral fracture syndrome accompanied by complications associated with radial nerve palsy. After the necessary investigation, he was diagnosed with a Holstein-Lewis fracture with radial nerve paralysis; he underwent open reduction internal fixation (ORIF), after which he was referred to physical therapy. Developing a successful postoperative rehabilitation program that consists mostly of functional physical therapy interventions is essential for the treatment of this condition. Outcome measures like the Numerical Pain Rating Scale (NPRS), Disabilities of the Arm, Shoulder, and Hand (DASH) score, and Patient-Rated Wrist Evaluation (PRWE) score were recorded before and after rehabilitation, and pain reduction, improvement in strength, range of motion (ROM), grip strength, and activities of daily living (ADL) were found. The purpose of this case report is to present a comprehensive treatment plan that includes ROM exercises, cryotherapy, and strengthening of grip using a robotic glove for a patient who had a wrist drop and underwent ORIF surgery. This tailored intervention was effective in speeding up the return of functional abilities and improving function in ADLs.

## Introduction

Holstein and Lewis discovered a unique kind of fracture of the humeral shafts with a specific propensity for radial nerve palsy. This fracture is situated within the distal one-third of the humeral shaft, presenting as a spiral type. The distal fragment of bone consistently exhibits proximal displacement, with its proximal end displaying radial deviation. The entrapment of the radial nerve occurs at the fracture site, and the presence of a comminuted fragment introduces a potential risk for nerve damage due to the oblique surface of the distal end of the proximal fragment [1]. The fracture line extends from the proximolateral to the distomedial plane. In accordance with the Orthopaedic Trauma Association (OTA) classification, humeral shaft fractures categorized as type 12A1.3 meet the criteria for classification as a Holstein-Lewis fracture, and the radial nerve is frequently injured together with the fracture line, which is in close proximity to the elbow joint at its distal end. The annual occurrence varies between 13 and 20 per 100,000 individuals and has been observed to increase with age [2]. The impact of the injury causes the proximal fragment to be pushed distally, which displaces the intermuscular septum and the radial nerve that is located within the septum's foramen. Concurrently, it causes the entrapment or laceration of the radial nerve between fragments of the bone. The incidence of radial nerve damage in adults with humeral shaft fractures varies from 7% to 17% [3].

The preferred treatment for this type of fracture or injury consists of primary open reduction and internal fixation (ORIF). Several surgical methods have been implemented to treat the fractures of the shaft humerus occurring at the distal third of the shaft. In most clinical settings, the posterior approach is favoured for surgical procedures [4,5]. The operative intervention has demonstrated enhanced reliability for immediate fracture stability, providing predictable alignment and enabling early elbow mobilization, although there are potential complications such as iatrogenic nerve damage, olecranon impingement, infection, and loosening of hardware [6]. The additional issue with such methods is that they can result in muscular weakness and restricted range of motion (ROM) due to injuries to the triceps brachii or brachioradialis. Moreover, regaining pre-injury functions, such as elbow motion, muscle strength, and grasping, is crucial [7]. It mostly affects the elbow and shoulder, which has an impact on the person's entire functional capacity. Therefore, the intent of physical therapy must be to enhance upper-limb function [8]. During the recovery stage, exercise and muscle retraining preserve joint ROM and enhance motor function recovery when muscle reinnervation occurs [9]. An efficient rehabilitation program is necessary to avoid joint stiffness and restore ROM following surgery for distal humerus intraarticular fractures [10].

The hallmark of a radial nerve injury is a wrist drop. The non-functioning wrist extensors are overpowered by flexor tone, causing the hand to flex [11]. Individuals with loss of hand function have significant challenges with gripping, grasping, and manipulating objects, which makes it difficult for them to carry out daily tasks on their own [12]. The hand extension robotic orthosis glove was designed through an iterative method to help people with major hand impairments. Portability, lightweight qualities, and ease of setup and use are highlighted in this design. The robotic apparatus is composed of a batting glove equipped with artificial tendons integrated into the fingers of the glove. A linear actuator pulls and pushes the tendons to flex and extend the finger [13]. In this case report, we see the case of a 20-year-old male with a Holstein-Lewis fracture associated with radial nerve palsy, treated with ORIF, and how physical therapy contributed to his improvement. Our study's goal was to assess the impact of using a robotic glove on early wrist mobility and grip strength in the case of a wrist drop related to a distal third humerus fracture.

## Case presentation

Patient information

We present a case of a 20-year-old male who experienced a road traffic accident and was brought to the emergency causality at our hospital. Having been hit by a four-wheeler, he had undergone an X-ray investigation that revealed a Holstein-Lewis fracture on the right side. He experienced severe pain in his right arm, which aggravated during movement, and was relieved with rest and medications. Pain was acute in onset, continuous, and non-radiating in nature. On observation, the shoulder was adducted with the elbow in 45-degree flexion, and the forearm was in a semi-prone position. A bony deformity was noted over the distal arm. He had no history of head trauma. The patient was managed with ORIF with plate osteosynthesis and radial nerve exploration under nerve block. After surgical repair, a tailor-made physical therapy regimen was started.

Clinical findings

The patient's consent was acquired before the examination. He was cooperative, conscious, and well-oriented toward people, places, and time. On clinical examination, the patient was afebrile and maintained hemodynamic stability. On observation, the arm was supported with the sling. The swelling was present on the distal arm. No previous surgical scars, discharging sinuses, or dilated veins were seen. On palpation, the local temperature was raised over the right arm. Grade 3 tenderness was noted over the midshaft humerus. Wrist and finger extension were absent; wrist and finger flexion were present. Upon examination, the patient demonstrated a right-sided wrist drop, exhibiting complete passive wrist joint ROM and restricted active and passive ROM in the shoulder for flexion, abduction, and internal and external rotation. The strength was notably diminished in the right upper limb. The patient reported experiencing intermittent and dull pain, with an activity-related pain rating of 7/10 and a pain rating of 3/10 at rest, as assessed using the Numerical Pain Rating Scale (NPRS).

Radiological findings

**Figure 1:**
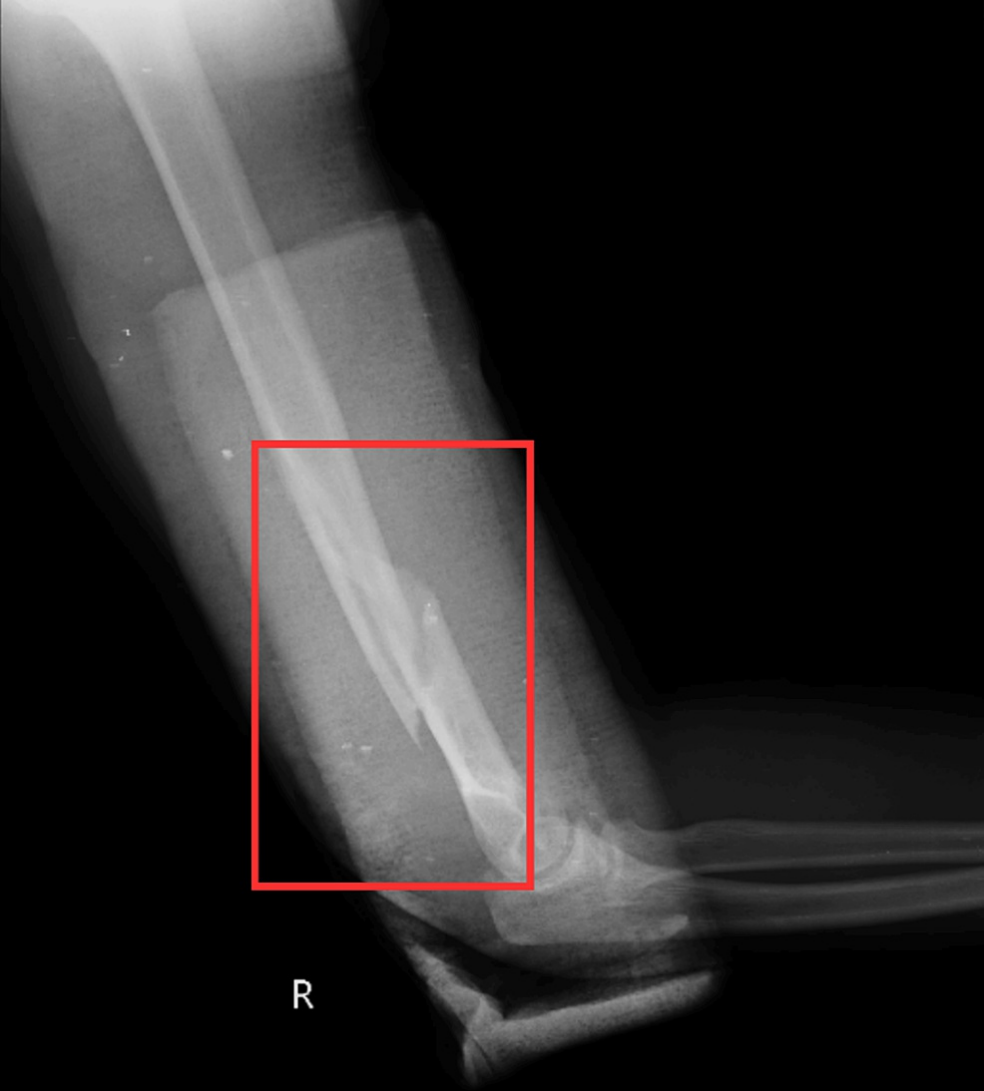
Preoperative X-ray of the arm (lateral view) The red rectangle indicates a distal 1/3rd humerus fracture (Holstein-Lewis fracture).

The patient underwent an investigational X-ray of the arm, which revealed a displaced distal 1/3rd humerus fracture, as shown in Figure 1.

**Figure 2:**
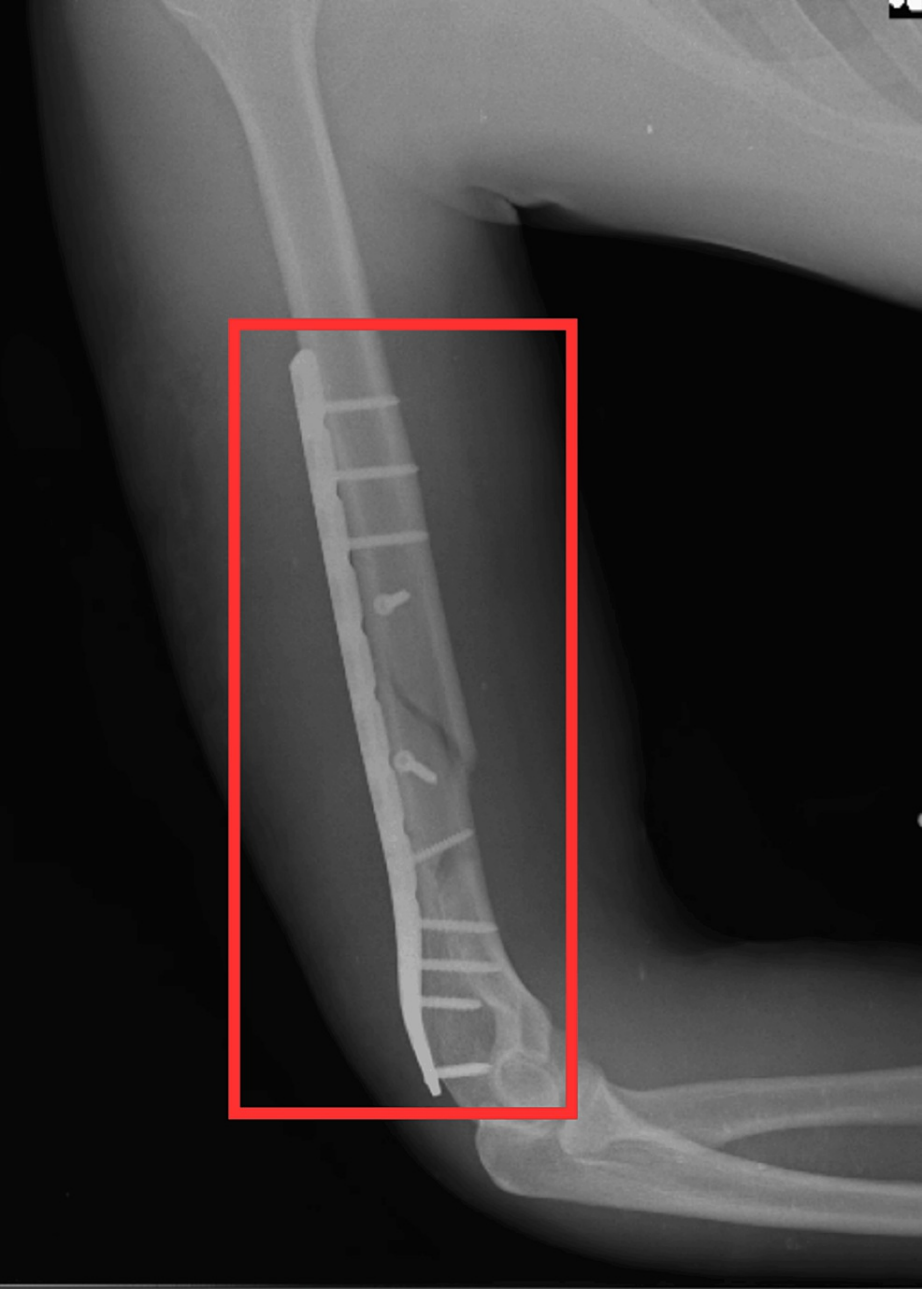
Postoperative X-ray (lateral view) The red rectangle indicates open reduction internal fixation of distal 1/3rd humerus fracture with plate osteosynthesis and nailing.

Figure 2 shows the lateral postoperative radiograph of the arm.

Therapeutic intervention

The physiotherapist designed tailored exercise sessions based on the patient's clinical status. Table 1 depicts the physical therapy protocol. On the second postoperative day, the patient started active, assisted physical therapy with elbow movements while wearing a dressing and a wide-arm polysling for comfort.

**Table 1:** Physiotherapy intervention ROM: range of motion; PROM: passive range of motion; AAROM: active assisted range of motion; AROM: active range of motion; reps: repetitions; PNF: proprioceptive neuromuscular facilitation; FES: functional electrical stimulation; D: diagonal

Week	Goal	Intervention	Regimen
Weeks 0-2 (during plaster cast)	To maintain the ROM of the forearm, wrist, digits, and scapula, reduce dependent oedema and stiffness.	AAROM to the forearm, PROM to the wrist, hand, scapular depression, protraction, and retraction exercises	10 reps x 1 set (2 times/ day)
	To reduce pain	Cryotherapy	7 mins, 2 times/day
	To maintain the strength and ROM of the unaffected limb	AROM exercises for unaffected limbs	10 reps x 1 set, twice daily
	Support to prevent joint contracture and muscle-tendon lengthening	Sling, cock-up splint	-
Weeks 2-6	Muscle re-education	FES with galvanic current, progressing to faradic current with voluntary effort	10 minutes
	To improve ROM and prevent stiffness	Active and active-assisted ROM to tolerance at shoulder, elbow, PROM to wrist, and hand with robotic gloves	10 reps x 1 set, twice daily
	To increase the strength of the parascapular, wrist, and hand muscles	Gentle isometric exercises, progression: isotonic exercises to the forearm, open kinetic chain exercises, Codman exercises	10 reps x 1 set, twice daily
	To enhance the strength of the wrist musculature	PNF D1 flexion and extension	10 reps x 1 set, twice daily
Weeks 6-12	To maintain ROM and prevent stiffness	Active ROM to the shoulder and elbow	10 reps x 1 set, twice daily
	To maintain the strength	Resistive exercises in open and close kinetic chain to shoulder elbow with elastic tubing and dumbbells	10 reps x 1 set, twice daily
	To maintain the ROM of the wrist joint and grip strength	Active ROM exercises and grasping activities with robotic gloves	10 reps x 1 set, twice daily

**Figure 3:**
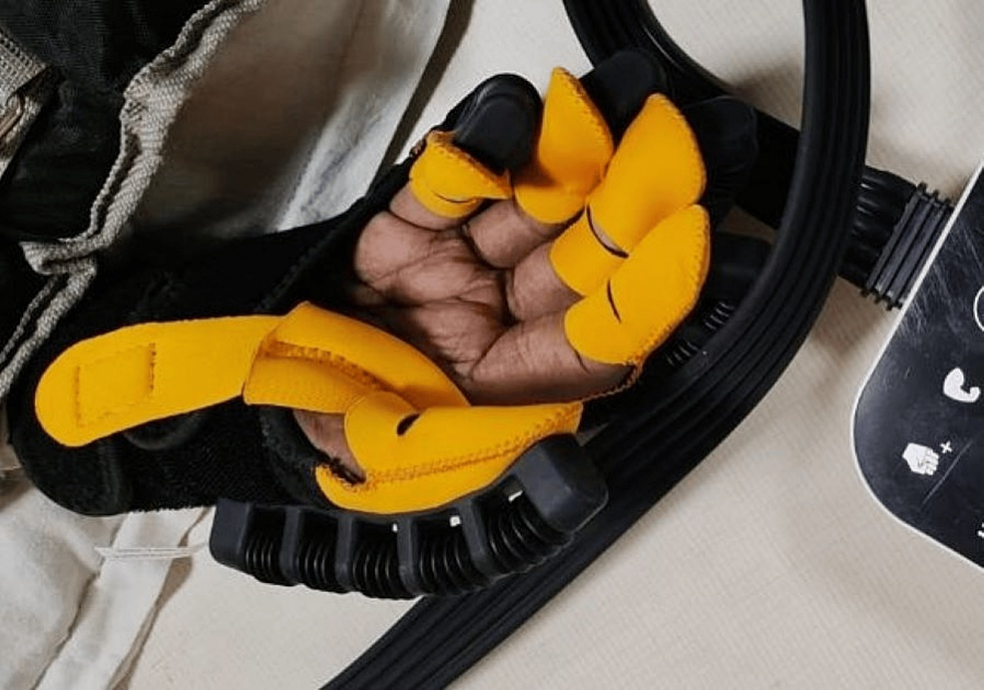
Patient performing wrist and finger flexion using robotic gloves

Figure 3 shows the patient performing wrist and finger flexion with the help of robotic gloves.

**Figure 4:**
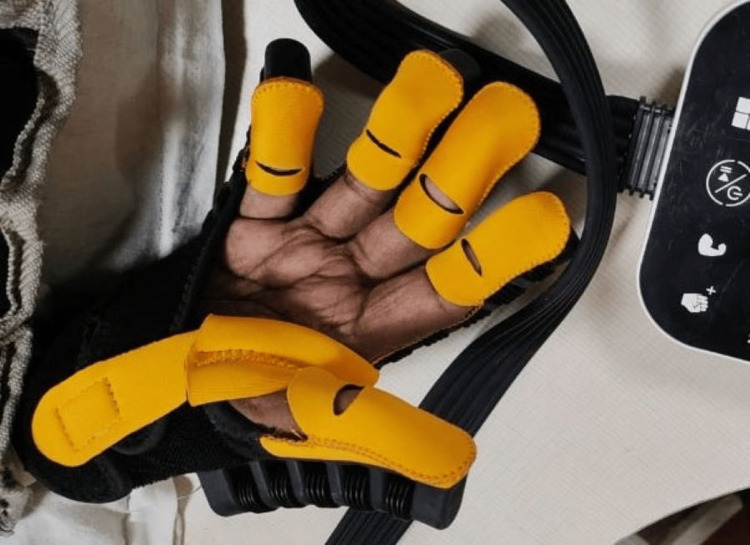
Patient performing wrist and finger extension using robotic gloves

Figure 4 shows the patient performing wrist and finger extension with the help of robotic gloves. 

Follow-up and outcome measures

For four weeks, the patient underwent a structured physical therapy regimen, followed by a subsequent follow-up evaluation. The findings of the manual muscle testing of the upper limb are shown in Table 2.

**Table 2:** Upper limb manual muscle testing (right side) 0: no contraction palpated; 3-: some but not complete range of motion against gravity; 4: complete range of motion against gravity with moderated resistance

Muscles	Pre-intervention	Post-intervention
Shoulder flexors	3-	4
Shoulder extensors	3-	4
Shoulder abductors	3-	4
Shoulder adductors	3-	4
Elbow flexors	3-	4
Elbow extensors	3-	4
Wrist flexors	3-	4
Wrist extensors	0	4

Table 3 depicts the ROM of the upper limb.

**Table 3:** Upper limb range of motion (in degrees, right side) N/A: not assessable

Movement	Pre-intervention	Post-intervention
Shoulder flexion	0-90	0-165
Shoulder extension	0-20	0-40
Shoulder abduction	0-90	0-160
Shoulder adduction	90-0	160-0
Elbow flexion	0-20	0-100
Elbow extension	20-0	100-0
Wrist extension	N/A	0-45
Wrist ulnar deviation	0-15	0-25
Wrist radial deviation	0-5	0-15

The pre-and post-treatment findings (right side) of the outcome measures are shown in Table 4.

**Table 4:** Outcome measures DASH: Disabilities of the Arm, Shoulder, and Hand; NPRS: Numerical Pain Rating Scale; PRWE: Patient-Rated Wrist Evaluation, N/A: not assessable

Outcome measures	Pre-intervention	Post-intervention
DASH score	N/A	45/100
NPRS	On activity-7/10; On rest-3/10	On activity-3/10; On rest-1/10
PRWE score	135/150	50/150

## Discussion

A Holstein-Lewis fracture represents a humeral shaft fracture with a characteristic spiral pattern, potentially leading to radial nerve injury, given its anatomical course around the humerus. Open reduction internal fixation is an ideal course of treatment to stabilize the fracture site, lower the likelihood of non-union, and promote a prompt return to everyday activities. A progressive physical therapy regimen can effectively regain the early restoration of elbow ROM. For wrist drops, a cock-up splint is employed, and physical therapy begins on the second postoperative day. A humerus fracture-related radial nerve palsy has a high incidence of spontaneous recovery [14]. Postoperative physical therapy care is essential in order to achieve an early functional recovery and avoid subsequent complications. In this paper, we have discussed a clinical case of a 20-year-old male diagnosed with a Holstein-Lewis fracture and managed surgically with ORIF with plate osteosynthesis and nailing. The ORIF led to postoperative pain, loss of ROM, strength, and wrist drop due to injury to the radial nerve for which the patient underwent physical therapy management, which proved to be effective in early functional recovery. Strengthening exercises, ROM exercises, stretching, and joint mobilization are beneficial in helping the person become functionally independent [15].

A study by Casmus et al. demonstrated that a structured rehabilitation program that incorporates proprioceptive exercises, plyometric ball activities, resistance tubing exercises, and dumbbell activities can effectively recover early ROM at the elbow joint [16]. A case study concluded that combining mirror therapy with conventional therapy after a humeral shaft fracture improves the function of the affected joint. Therefore, it should be included in the fracture rehabilitation regimen [8]. The hand and fingers are vital anatomical structures for executing a wide range of functional activities in everyday life, especially for grasping and handling objects. Correia et al. demonstrated findings that indicate that individuals experiencing compromised hand function attributable to spinal cord injury demonstrated significant enhancements in power and pinch grip forces, alongside improvements in the active ROM of the fingers, facilitated by glove assistance [17]. The robotic exoskeletal glove emerges as a viable assistive technology or adjunct rehabilitative intervention to maximize sensorimotor deficits and upper limb and hand-related functional capacities for those who have hand paresis or paralysis after a stroke [18]. Radial nerve damage from a humerus fracture can result in serious and irreversible disability. Fader et al. found that the strategic application of synergistic upper extremity movement patterns in multiple planes, incorporating neuromuscular irradiation or overflow, and utilizing neuroplasticity led to enhanced strength and ROM [19]. Similarly, we used proprioceptive neuromuscular facilitation and found positive effects. We gave cryotherapy to reduce postoperative pain. According to Khadijah et al., a distinct form of transcendence that is specifically attained by using cold compresses is a change in the way that pain is perceived, with an increased focus on the sensation of cold, which enhances one's comfort. Based on current theories and research, it may be concluded that cryotherapy is preferable to warm compresses in terms of reducing pain perception and improving comfort [20].

## Conclusions

An integral component of the therapeutic approach for Holstein-Lewis fracture is determining the optimal postoperative rehabilitation plan that emphasizes elements of functional physiotherapy. Meticulously designed rehabilitation protocols lead to better functional results after surgery. Wearable hand rehabilitation apparatus can help physiotherapists improve early fine motor movement, wrist mobility, grip strength, and the effectiveness of rehabilitation exercises. Physiotherapeutic interventions, encompassing cryotherapy, ROM exercises employing robotic gloves, and muscle-strengthening exercises, prove advantageous for patients with Holstein-Lewis fractures.
